# Regulatory networks in plant responses to drought and cold stress

**DOI:** 10.1093/plphys/kiae105

**Published:** 2024-03-21

**Authors:** June-Sik Kim, Satoshi Kidokoro, Kazuko Yamaguchi-Shinozaki, Kazuo Shinozaki

**Affiliations:** RIKEN Center for Sustainable Resource Science, 1-7-22 Suehiro-cho, Tsurumi-ku, Yokohama, 230-0045Japan; Institute of Plant Science and Resources, Okayama University, 2-20-1 Chuo, Kurashiki, 710-0046Japan; School of Life Science and Technology, Tokyo Institute of Technology, 4259 Nagatsuta-cho, Midori-ku, Yokohama, 226-8502Japan; Research Institute for Agriculture and Life Sciences, Tokyo University of Agriculture, 1-1-1 Sakuragaoka, Setagaya-ku, Tokyo, 156-8502Japan; Graduate School of Agriculture and Life Science, The University of Tokyo, 1-1-1 Yayoi, Bunkyo-ku, Tokyo, 113-0032Japan; RIKEN Center for Sustainable Resource Science, 1-7-22 Suehiro-cho, Tsurumi-ku, Yokohama, 230-0045Japan; Institute for Advanced Research, Nagoya University, Furo-cho, Chikusa-ku, Nagoya, 464-8601Japan

## Abstract

Drought and cold represent distinct types of abiotic stress, each initiating unique primary signaling pathways in response to dehydration and temperature changes, respectively. However, a convergence at the gene regulatory level is observed where a common set of stress-responsive genes is activated to mitigate the impacts of both stresses. In this review, we explore these intricate regulatory networks, illustrating how plants coordinate distinct stress signals into a collective transcriptional strategy. We delve into the molecular mechanisms of stress perception, stress signaling, and the activation of gene regulatory pathways, with a focus on insights gained from model species. By elucidating both the shared and distinct aspects of plant responses to drought and cold, we provide insight into the adaptive strategies of plants, paving the way for the engineering of stress-resilient crop varieties that can withstand a changing climate.

## Introduction

The substantial rise in atmospheric carbon dioxide levels has contributed to global warming, adversely affecting ecological conditions. This surge is intensifying climate change, leading to frequent droughts, severe temperature swings, and rising soil salinity, all of which detrimentally affect ecosystem health and agricultural output. Indeed, plant growth is greatly affected by water-limited conditions such as drought and salinity, as well as extreme temperature conditions. However, land plants have evolved mechanisms that allow them to withstand these stressors and sustain survival.

Early research on plant responses to abiotic stress focused on the underlying physiology and biochemistry using the techniques available at the time (Iljin 1957; Lyons 1973). Since the 1980s, the focus has shifted to obtaining a sophisticated understanding of the genetic mechanisms behind these responses, driven by continuous technical advances and the adoption of Arabidopsis (*Arabidopsis thaliana*) as a model species. The complete sequencing of the Arabidopsis genome in 2000 (The Arabidopsis Genome Initiative 2000) accelerated the identification of genes activated in response to various stresses, including cold and drought, revealing both significant overlap in gene networks and distinct pathways for each stress type (Maruyama et al. 2012; Zhu 2016; Waadt et al. 2022). Over the past decade, next-generation sequencing has further advanced this field. RNA-seq has become a crucial tool for transcriptome analysis, providing detailed and digitized global expression profiles and allowing the comparison of even subtle changes in expression (Wang et al. 2014). Concurrently, DNA-seq has hastened the creation of reference genomes for a growing number of plant species, facilitating the construction of orthologous groups of functional genes to broaden insights from model species to other plants (Nguyen et al. 2019). The accumulated knowledge is being harnessed to develop stress-resilient crops through transgenic DNA technology and genome editing (Bailey-Serres et al. 2019; Hickey et al. 2019; Cooper and Messina 2023).

**Figure 1.**
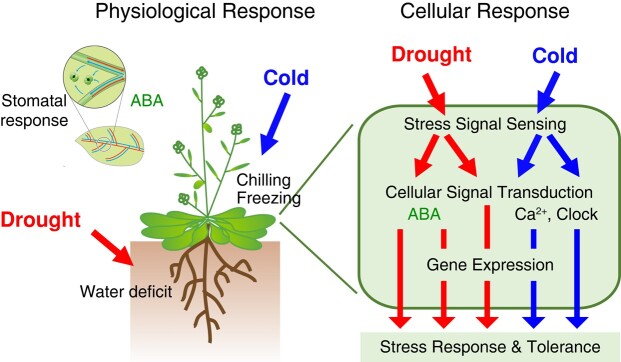
Physiological and cellular responses to drought and cold stress in plants. Under severe drought and cold stress conditions, plants alter their physiology to acquire stress tolerance. During drought stress, ABA plays a crucial role in stomatal closure, which limits water loss. By contrast, cold stress is characterized by chilling or freezing, which severely disrupts plant growth. However, plants have evolved mechanisms to survive these stresses. During cellular stress responses, plant cells directly sense drought and cold stress or receive systemic signals from other parts of the plant. Through multiple interconnected signaling cascades, these stress signals activate the expression of genes specific to each type of stress. These genes encode proteins that enhance plant stress tolerance.

Plant drought and cold response pathways comprise highly complex sensing, signaling, and response mechanisms specific to each type of stress (Fig. 1). As the 2 stresses do not often co-occur, the pathways for each stress are essentially independent. Drought responses are largely driven by biosynthesis of and signaling associated with the phytohormone abscisic acid (ABA), with plants utilizing root-to-shoot signaling to adapt to dry conditions of varying intensities and durations. Responses to chilling and freezing are more uniform, since temperature shifts are generally simultaneously perceived across plant shoots and are connected with diurnal, circadian, and annual rhythms. However, the molecular functions of cold- and dehydration-responsive genes significantly overlap. Angiosperms such as Arabidopsis, soybean (*Glycine max*), and rice (*Oryza sativa*) induce genes encoding late embryogenesis abundant (LEA) proteins and molecular chaperones during both drought and cold stresses, while genes related to photosynthesis are typically downregulated (Maruyama et al. 2012). Cold affects plant physiology via temperature-related effects, whereas freezing-induced damage primarily results from dehydration associated with the formation of ice crystals (Thomashow 1999). Thus, plants have developed a unified strategy of gene regulation to counter the effects of high osmolarity during both drought and cold stress (Thomashow 1999; Yamaguchi-Shinozaki and Shinozaki 2006; Zhu 2016).

In this review, we summarize milestone discoveries and recent advances in our understanding of drought and cold stress responses and tolerance, with a primary focus on regulatory systems including gene expression, signaling, and perception. We also discuss notable applications of recent technological advancements in crop breeding. The term “plant” refers to terrestrial angiosperms throughout this review unless otherwise specified.

## Plant responses to drought stress

Water scarcity during drought poses significant challenges to plants. Dehydration, resulting from high osmolality or drought stress, leads to a loss of turgidity and pressure against the cell wall, both essential for maintaining plant structure and growth. Energy production falters as photosynthesis rates drop, causing a metabolic imbalance and the accumulation of harmful compounds. The phytohormone ABA plays a crucial role in drought responses, minimizing water loss through stomatal closure and initiating protective mechanisms against water scarcity (Finkelstein 2013). Drought- and ABA-inducible genes are integral to these protective actions, encoding LEA proteins, chaperones, enzymes for ABA and osmoprotectant biosynthesis, sugar and proline transporters, aquaporins, and detoxification enzymes to neutralize reactive oxygen species (ROS) (Yamaguchi-Shinozaki and Shinozaki 2006; Urano et al. 2009; Zhu 2016; Yoshida and Fernie 2023). *RESPONSIVE TO DEHYDRATION29A* (*RD29A*) and *RD29B*, early identified Arabidopsis genes for LEA proteins, are widely used as stress markers in research due to their sensitive induction by ABA and drought (Yamaguchi-Shinozaki et al. 1992). Other genes inducible by cold or drought stress, such as galactinol synthase (GolS) genes, lead to the production of raffinose and galactinol—soluble carbohydrates that protect against osmotic damage (Taji et al. 2002). Drought-responsive transcription factors (TFs) regulate these stress-protective genes and are modulated by stress signals from ABA and other signaling pathways.

### Biosynthesis, catabolism, and transport of ABA in response to drought stress

Endogenous ABA levels increase when plants encounter water-limited conditions, leading to systemic changes that protect them against the stress. The well-established ABA biosynthesis pathway involves catalytic enzymes, including NINE-CIS-EPOXYCAROTENOID DIOXYGENASE (NCED), ABA DEFICIENT2 (ABA2), and ARABIDOPSIS ALDEHYDE OXIDASE3 (AAO3) (Hauser et al. 2011; Finkelstein 2013). NCED, originally identified as Viviporous14 in maize (*Zea mays*), is the rate-limiting enzyme of this pathway (Tan et al. 1997), with notable induction by drought first observed in cowpea (*Vigna unguiculata*) (Iuchi et al. 2000). Of the 5 NCED isoforms in Arabidopsis, *NCED3* transcripts show rapid drought-responsive accumulation (Iuchi et al. 2001; Tan et al. 2003). Loss-of-function *nced3* mutants have reduced endogenous ABA levels, with impaired stomatal closure and plant survival (Iuchi et al. 2001). Other isoforms (such as *NCED5*, *NCED6*, and *NCED9*) are primarily expressed in developing seeds, contributing to seed maturation and dormancy (Martínez-Andújar et al. 2011; Frey et al. 2012).

ABA catabolism maintains ABA homeostasis. Within the cytochrome P450 monooxygenase family 707 (CYP707), a specific subclade of ABA 8′-hydroxylases comprising CYP707A1 to CYP707A4 plays a pivotal role in Arabidopsis (Krochko et al. 1998; Kushiro et al. 2004; Saito et al. 2004). These 4 members collectively regulate seed dormancy and germination, exhibiting inducible gene expression in response to dehydration stress and subsequent rehydration (Kushiro et al. 2004; Saito et al. 2004; Okamoto et al. 2006; Umezawa et al. 2006). The drought-responsive expression of *CYP707A* and *NCED3* genes underscores the precise control of ABA levels through the maintenance of the delicate balance between biosynthesis and catabolism. Moreover, ABA inactivation occurs through conjugation with oxidative catabolites. Notably, ABA glucosyl ester (ABA-GE) has been identified in various tissues of different plant species (Piotrowska and Bajguz 2011). β-Glucosidases hydrolyze ABA-GE to release unconjugated ABA from the endoplasmic reticulum (Dietz et al. 2000; Lee et al. 2006). Hence, this pathway likely serves as a rapid ABA release mechanism for an immediate response to drought before the full activation of ABA biosynthesis.

The preferential expression of genes encoding key ABA biosynthesis enzymes including NCED3, ABA2, and AAO3 has been observed in leaf vascular tissues, where water availability is sensed and through which ABA is transported in the plant (Koiwai et al. 2004; Endo et al. 2008; Kuromori et al. 2014). Several transporters involved in ABA trafficking across cellular membranes have been identified (Kuromori et al. 2018). These transporters belong to families such as the ATP-binding cassette (ABC) subfamily, Nitrate transporter1/Peptide transporter family (NPF), and Detoxification efflux carrier (DTX)/Multidrug and toxic compound extrusion family. In particular, Arabidopsis *ABCG25* exhibits ABA- and drought-inducible expression and encodes an ABA exporter restricted to vascular tissues (Kuromori et al. 2010). *ABCG40* shows strong expression in guard cells and facilitates ABA uptake to promote ABA-mediated stomatal closure (Kang et al. 2010). Among the other transporter families, *NPF4.6* is an ABA importer gene predominantly expressed in inflorescence stems and roots (Kanno et al. 2012). In addition, *DTX50* is strongly expressed in rosette leaf vasculature, facilitating ABA-responsive stomatal closure through ABA efflux (Zhang et al. 2014).

### ABA perception complex and drought signaling

**Figure 2.**
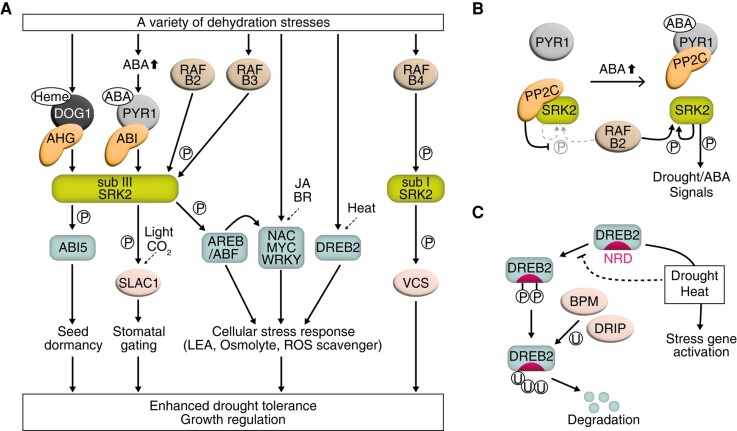
Molecular machinery of drought stress perception, signaling, and responses in vascular plants. **A)** Overview of ABA–dependent and –independent drought signaling pathways. In the ABA-dependent pathway, the ABA-bound receptor complex modulates drought signaling via protein phosphorylation (circled P). ABA signal transduction induces stress responses including seed maturation and dormancy, stomatal gating, and drought-responsive gene expression. There are also ABA-independent branches of drought stress signaling, including the heme-bound DOG1–AHG pathway and DREB2-mediated transcriptional regulation. B-type RAFs are recently discovered components of drought signaling. JA, jasmonic acid; BR, brassinosteroid. **B)** The ternary complex model of ABA perception consists of the receptor (PYR1/PYL/RCAR), the signal activator (subclass III SnRK2/SRK2), and the suppressor (PP2C). B2-type RAFs are recently discovered components that constitutively phosphorylate SnRK2s. **C)** Post-translational modifications of DREB2 in response to stress. Under normal conditions, the nascent DREB2 protein is phosphorylated within its NRD domain and is subsequently targeted for proteasomal degradation via ubiquitination (circled U) mediated by specific E3 ligases (BPMs and DRIPs). Drought and heat stress prevent DREB2 phosphorylation, thereby enhancing its stability and allowing it to activate downstream gene expression.

The established model for ABA perception and signaling in vascular plants involves a series of protein phosphorylation cascades mediated by 3 key components: the ABA receptors PYRABACTIN RESISTANCE1 (PYR1) and PYR1-LIKE (PYR1/PYL, also named REGULATORY COMPONENT OF ABA RECEPTOR [RCAR]), the signaling activator SUCROSE NON-FERMENTING1-RELATED PROTEIN KINASE2 (SnRK2 or SRK2), and the suppressor TYPE 2C PROTEIN PHOSPHATASE (PP2C) (Cutler et al. 2010; Finkelstein 2013) (Fig. 2, A and B).

The discovery of the PYR1/PYL/RCAR family of ABA receptors marked a significant breakthrough in understanding ABA signaling in plants. In Arabidopsis, this family has 12 functionally redundant members, with the prominent PYR1/RCAR11 being the first receptor found to bind ABA (Ma et al. 2009; Park et al. 2009; Santiago et al. 2009). Upon binding to ABA, the receptors PYR1/PYLs/RCAR interact with PP2Cs and inhibit their activity, enabling the phosphorylation and activation of SnRK2s, thereby initiating ABA signaling (Melcher et al. 2009; Miyazono et al. 2009; Nishimura et al. 2009). The search for additional receptor ligands for agricultural applications has identified several ABA analogs that exhibit stronger sensitivity and greater receptor selectivity, such as quinabactin (Okamoto et al. 2013) and opabactin (Vaidya et al. 2019).

SnRK2s phosphorylate target proteins, including themselves, and their catalytic activity requires their own phosphorylation (Yoshida et al. 2002; Belin et al. 2006; Soma et al. 2023). The Arabidopsis SnRK2/SRK2 family consists of 10 isoforms of 3 subclasses based on protein homology (Mustilli et al. 2002; Boudsocq et al. 2004). Three SnRK2 isoforms, SnRK2.2/SRK2D, SnRK2.3/SRK2I, and SnRK2.6/SRK2E/OPEN STOMATA1 (OST1), belong to subclass III and redundantly mediate most ABA signal transduction. The triple loss-of-function mutant of subclass III SnRK2 members lacks ABA-responsive stomatal closure, seed maturation, and dormancy and exhibits no ABA-mediated induction of most drought-responsive genes (Fujii and Zhu 2009; Fujita et al. 2009; Nakashima et al. 2009; Umezawa et al. 2009). OST1 is specifically involved in regulating stomatal closure, as its ABA-induced phosphorylation activates the channel SLOW ANION CHANNEL-ASSOCIATED1, which induces anion efflux from guard cells, resulting in stomatal closure (Mustilli et al. 2002; Geiger et al. 2009). This pathway is also required for other stimuli that induce stomatal closure, such as CO_2_ and the phytohormone jasmonate (JA) (Hsu et al. 2018; Munemasa et al. 2019). By contrast, ABA treatment failed to induce or activate any of the 4 subclass I–type SnRK2s (SnRK2.1/SRK2G, SnRK2.4/SRK2A, SnRK2.5/SRK2H, and SnRK2.10/SRK2B), which are rapidly activated by osmotic stress without stress-responsive ABA accumulation (Boudsocq et al. 2004; Yoshida et al. 2006). Subsequent studies unveiled their ABA-independent role under drought stress: subclass I–type SnRK2s redundantly target and activate the mRNA decapping activator VARICOSE in cytosolic processing (P)-bodies, promoting stress-responsive mRNA decay to enhance stress acclimation (Soma et al. 2017; Kawa et al. 2020).

Arabidopsis harbors dozens of PP2C isoforms, with those in subclade A being key players in ABA signaling (Schweighofer et al. 2004), including ABA INSENSITIVE 1 (ABI1) and ABI2 (Koornneef et al. 1984). In the absence of ABA, ABI1 interacts with and dephosphorylates subclass III–type SnRK2s, preventing their activation (Gosti et al. 1999; Yoshida et al. 2006). PP2C subclade A comprises 9 members, including 2 ABIs. At least 6 of these PP2Cs (ABI1, ABI2, HYPERSENSITIVE TO ABA1 [HAB1], HAB2, ABA HYPERSENSITIVE GERMINATION1 [AHG1], and AHG3) target and dephosphorylate the SnRK2s, albeit with slight differences in their physiological roles. Two HABs share similar functions with ABI (Umezawa et al. 2009; Vlad et al. 2009). AHG1 and AHG3 are involved in seed-related responses, promoting and maintaining dormancy through both PYR1/PYL/RCAR-dependent and -independent mechanisms. The eukaryote-conserved heme-binding protein DELAY OF GERMINATION1 (DOG1) plays a critical role in Arabidopsis seed dormancy and the adaptive control of germination (Graeber et al. 2014). Heme-bound DOG1 interacts with AHG1, preventing this PP2C from binding to PYR1/PYLs/RCARs and inactivating subclass III–type SnRK2s (Nishimura et al. 2018). A similar regulatory mechanism was established for AHG3, which is also modulated by ABA-bound PYR1/PYLs/RCARs (Née et al. 2017). The remaining subclade A PP2C members are 3 homologs: HIGHLY ABA-INDUCED1 (HAI1), HAI2, and HAI3. The genes encoding these subclade members exhibit ABA-inducible expression, and the *hai* triple mutant displays hypersensitivity to ABA (Fujita et al. 2009; Bhaskara et al. 2012). Thus, HAIs likely function in the negative feedback regulation of ABA signaling, although their target proteins and underlying mechanisms remain unclear (Chong et al. 2019).

### MAP kinases in ABA and drought signaling

Although subclass III SnRK2s are likely self-activated by autophosphorylation after their release from PP2C-imposed inhibition, an earlier discovery in the bryophyte *Physcomitrium patens* shed light on the involvement of the B-type MAP KINASE KINASE KINASE (M3K) subgroup of Raf-like kinases in SnRK2 activation under drought stress (Saruhashi et al. 2015). B-type Raf-like kinases are divided into 4 subfamilies (B1–B4). Four B3-type kinases (RAF3/M3Kδ1, RAF4/M3Kδ7, RAF5/M3Kδ6, and RAF6/M3Kδ5) phosphorylate and activate subclass III SnRK2s under osmotic stress in Arabidopsis (Katsuta et al. 2020; Lin et al. 2020; Takahashi et al. 2020). Through mutant analysis, 4 B2-RAFs (RAF7, RAF10, RAF11, and RAF12), in addition to B3-RAFs, were identified as activators of subclass III SnRK2s. Phosphorylation by these B2-RAFs and B3-RAFs is essential for the activation of subclass III SnRK2s (Lin et al. 2020). A recent study showed that B2-RAFs are constitutively active and activate subclass III SnRK2s when released from PP2C-imposed inhibition, whereas B3-RAFs are activated by stress (Soma et al. 2023). These findings indicate that autophosphorylation of subclass III SnRKs is not sufficient for ABA responses and that B2-RAFs are essential for subclass III SnRK2 activation in response to ABA, whereas B3-RAFs enhance subclass III SnRK2 activity under drought. By contrast, 3 B4-RAFs (RAF18, RAF20, and RAF24) activate the ABA-insensitive subclass I–type SnRK2s under dehydration conditions (Soma et al. 2020). The upstream signaling mechanisms for the B3-Raf-like and B4-Raf-like kinases remain unclear, leaving open questions about how plants sense drought stress.

### AREB/ABF transcription factors function in ABA-dependent gene expression

During plant drought responses, ABA signaling elicits the induction of numerous drought-responsive genes (Fig. 2A). The most highly conserved *cis*-acting element detected in the promoters of these genes is the ABA-RESPONSIVE ELEMENT (ABRE), a variant of the G-box (or E-box) element with an AGCT core (Marcotte et al. 1989; Mundy et al. 1990). The G-box sequence is preferentially recognized by basic leucine zipper (bZIP)-type TFs. Arabidopsis contains 78 bZIPs classified into 13 subgroups, with subgroup A encompassing the ABRE-BINDING PROTEIN/ABRE-BINDING FACTOR (AREB/ABF) members that play central roles in regulating ABA-responsive gene expression (Dröge-Laser et al. 2018). Among the AREBs/ABFs, AREB1/ABF2 and its 3 homologs (AREB2/ABF4, ABF1, and ABF3) regulate ABA- and drought-responsive gene expression in vegetative tissues by activating promoters harboring the ABRE (Choi et al. 2000; Uno et al. 2000). The activity of these TFs is induced by subclass III–type SnRK2-mediated phosphorylation (Fujita et al. 2005; Furihata et al. 2006). These 4 AREB/ABF members are functionally redundant, as evidenced by the more severe phenotypes observed in higher-order mutants compared with single *areb1* mutants (Fujita et al. 2005; Yoshida et al. 2010, 2015). Transcriptome analysis of quadruple *areb/abf* mutants revealed that these 4 members collectively regulate a major portion of ABA-responsive genes under the control of subclass III–type SnRK2s (Yoshida et al. 2015).

ABI5 is another AREB/ABF member that functions exclusively in seed dormancy and early seedling development. Germination in *abi5* mutants is insensitive to ABA treatment, whereas *ABI5*-overexpressing plants exhibit hypersensitivity to ABA (Finkelstein and Lynch 2000; Lopez-Molina et al. 2001). Similar to AREB1/ABF2, ABI5 TF activity requires phosphorylation mediated by subclass III–type SnRK2s (Nakashima et al. 2009; Nishimura et al. 2018). *ABI5* expression is induced by ABA, and ABI5 promotes seed dormancy by suppressing the expression of genes involved in germination, including those related to gibberellin biosynthesis and the production of seed coat mucilage (Finch-Savage and Leubner-Metzger 2006).

### DREB transcription factors function in ABA-independent gene expression

Drought-responsive ABA-independent gene regulatory pathways also exist in plants (Fig. 2A). Initially, the DEHYDRATION-RESPONSIVE ELEMENT (DRE) *cis*-acting element was identified from a set of dehydration-responsive genes expressed in leaves (Yamaguchi-Shinozaki and Shinozaki 1994). The same promoter motif was also reported in *COLD-REGULATED* (*COR*) genes and was named C-REPEAT (CRT) (Baker et al. 1994; Jiang et al. 1996). DRE/CRT is thus involved in cold stress–induced gene induction but does not function in ABA-responsive gene expression. Two subclades of APETALA2/ETHYLENE-RESPONSIVE FACTOR (AP2/ERF) TFs induce gene expression by targeting promoters containing DRE/CRT motifs: DRE-BINDING PROTEIN1/CRT-BINDING FACTOR (DREB1/CBF) and DREB2 (Stockinger et al. 1997; Liu et al. 1998; Shinwari et al. 1998). The DREB1/CBF family members are primarily recognized as cold-responsive regulators of gene expression with prominent roles in the chilling response (Liu et al. 1998). We delve further into their regulation and molecular functions in cold responses below.

The DREB2 subclade features a prominent and extensively characterized member, DREB2A. *DREB2A* is expressed differently in response to dehydration and heat stress: heat stress triggers a relatively short and strong induction, whereas drought stress leads to prolonged *DREB2A* transcript accumulation (Sakuma et al. 2006b). These distinct expression patterns involve 2 discrete regions in the *DREB2A* promoter, each independently inducing expression through different upstream TFs (Yoshida et al. 2008; Kim et al. 2011). Moreover, the post-translational regulation of DREB2A is crucial for its TF activity (Fig. 2C). DREB2A is highly unstable due to the presence of a serine/threonine-rich negative regulatory domain (NRD) located in the center of the protein (Sakuma et al. 2006a). The NRD undergoes extensive phosphorylation under normal growth conditions (Mizoi et al. 2019), leading to DREB2A degradation, which is facilitated by the ubiquitin E3 ligases BTB/POZ AND MATH DOMAIN (BPM) and DREB2-INTERACTING PROTEIN (DRIP) (Qin et al. 2008; Morimoto et al. 2017). However, heat and dehydration prevent these post-translational modifications, resulting in increased DREB2A stability (Morimoto et al. 2017; Mizoi et al. 2019). Additionally, heat stress–induced SUMOylation targeting the NRD inhibits the DREB2A–BPM interaction (Wang et al. 2020b). Notably, a similar BPM-mediated decay pathway has been identified for other drought-signaling components, including ABI1 (Julian et al. 2019) and HOMEOBOX PROTEIN6 (HB6) (Lechner et al. 2011), indicating the existence of an intricate protein turnover and signal interaction system that facilitates efficient drought responses in plants. Alongside the transcriptional repressor acting on *DREB2A* expression under normal growth conditions (Kim et al. 2012), these multi-level regulatory mechanisms indicate that plants use DREB2A to handle severe drought stress while also minimizing its negative effects on vegetative growth and development (Sakuma et al. 2006a; Qin et al. 2008).

### Other transcription factors and phytohormones involved in drought-responsive gene expression

The intricate mechanism of plant response to drought features plant-specific TFs that modulate drought-responsive gene expression (Fig. 2A). Members of the NAC (NAM, ATAF1/2, and CUC) family include STRESS-RESPONSIVE NACs (SNACs) that mediate the expression of abiotic stress–inducible genes (Tran et al. 2004). Among SNACs, ANAC072 (also known as RD26) and its functionally redundant homologs enhance the expression of many drought-responsive genes and regulate ABA-responsive leaf senescence (Fujita et al. 2004; Takasaki et al. 2015; Kamranfar et al. 2018). Similarly, ANAC096 is another SNAC that contributes to plant drought responses by physically interacting with members of the AREB/ABF family (Xu et al. 2013). Additionally, the plant-specific WRKY family is involved in a variety of plant stress responses (Eulgem et al. 2000). Several WRKY family members play roles in drought and ABA signaling. For example, Arabidopsis WRKY63 targets the *AREB1/ABF2* gene and enhances its ABA-inducible expression (Ren et al. 2010). By contrast, a subgroup comprising WRKY18, WRKY40, and WRKY60 represses multiple TFs involved in drought signaling, including AREB1/ABF2 and ABI5 (Shang et al. 2010). Drought- and ABA-inducible *HB6* encodes a TF that negatively regulates ABA-mediated drought responses, creating a feedback loop for efficient and flexible stress adaptation (Himmelbach et al. 2002).

Research on plant drought responses has increasingly focused on crosstalk with phytohormone pathways beyond ABA, although only a few genetic intersections have been established so far (Zhu 2016; Waadt et al. 2022; Yoshida and Fernie 2023). Arabidopsis MYC2, also known as JASMONATE INSENSITIVE1, is a key regulator of JA-responsive gene expression (Lorenzo et al. 2004). MYC2 and its cooperating TF MYB2 were originally identified as being responsible for ABA- and drought-inducible *RD22* expression (Abe et al. 1997, 2003). *MYC2* and *MYB2* also show ABA- and drought-inducible expression, suggesting that certain genetic interactions occur between JA signaling and the drought response (Abe et al. 2003). ANAC072/RD26 modulates brassinosteroid (BR) signaling as well by interacting with the essential BR-responsive TF BRASSINAZOLE RESISTANT1 (BZR1), thus influencing the induction of BR-responsive genes (Ye et al. 2017). Conversely, the BR-signaling kinase BRASSINOSTEROID-INSENSITIVE2, a repressor of BZR1 activity, activates ABA signaling by targeting SnRK2s (Cai et al. 2014). DEEPER ROOTING1 (DRO1) is a plasma membrane (PM)–localized protein of uncertain function, which was initially discovered in rice for its contribution to drought tolerance by altering root developmental patterns (Uga et al. 2013). DRO1 in angiosperms shows auxin-responsive gene expression and modifies root architecture to make the roots extend more deeply into the soil, thereby enhancing drought tolerance through improved water uptake (Guseman et al. 2016).

### Osmotic sensors and long-distance signaling in drought stress responses

**Figure 3.**
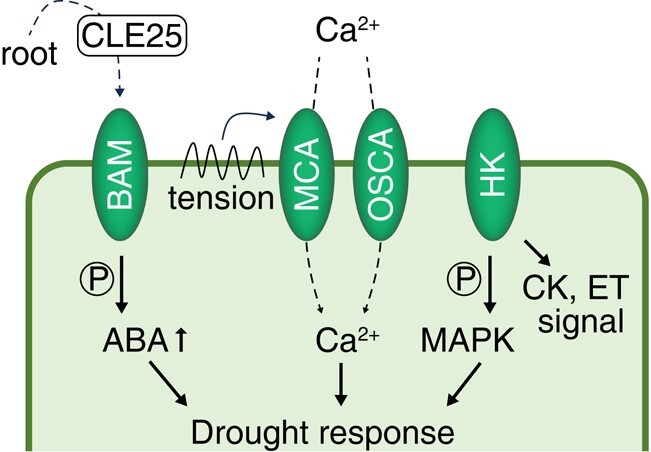
The osmosensor machinery in plants. HK, a homolog of the yeast osmosensor protein Sln1p, activates plant stress responses via MAP kinase (MAPK) signaling. HK also participates in cytokinin (CK) and ethylene (ET) signaling. Two BAM family members (BAM1 and BAM3) form a receptor complex for small CLE25 peptides, conveying the drought stress signal from the root. MCA and OSCA family members are permeable calcium ion (Ca^2+^) channels whose activation triggers Ca^2+^ uptake and associated stress responses.

In nature, plants encounter drought conditions with spatiotemporal heterogeneity, requiring signaling mechanisms to sense and communicate the stress throughout the plant (Fig. 3). Arabidopsis HISTIDINE KINASE1 (AHK1) is a PM-localized protein that is homologous to the yeast (*Saccharomyces cerevisiae*) osmosensor protein Sln1p (Urao et al. 1999). Indeed, ectopic expression of *AHK1* enhanced plant survival under drought (Tran et al. 2007; Wohlbach et al. 2008). Close homologs of AHK1 also function in cytokinin signaling, making it challenging to determine their specific roles as osmosensors (Jeon et al. 2010; Nishiyama et al. 2011). A recent study in the moss *P. patens* revealed that ethylene receptor–related HKs activate SnRK2s via the activation of RAF kinase, providing additional insights into plant HK-mediated osmosensing mechanisms (Toriyama et al. 2022). Calcium ion (Ca^2+^) channels are also candidate osmosensors. The Ca^2+^-permeable mechanosensitive channels MID1-COMPLEMENTING ACTIVITY1 (MCA1) and MCA2 sense cell wall tension in Arabidopsis, facilitating adaptive growth under dehydration conditions (Nakagawa et al. 2007; Yoshimura et al. 2021).

Both directionless diffusion and directional transduction occur during systemic signaling arising from stressed tissues. ROS and Ca^2+^ influence ABA signaling and stomatal closure (Pei et al. 2000; Waszczak et al. 2018). RESPIRATORY BURST OXIDASE HOMOLOG D (RBOHD), a key component of the systemic ROS wave, is activated by local ROS waves and subsequent Ca^2+^ accumulation. RBOHD then triggers another ROS burst that is transferred to neighboring cells (Suzuki et al. 2013). In guard cells, the ROS–Ca^2+^ signal cooperates with ABA to stimulate stomatal closure, triggering plant stress acclimation (Suzuki et al. 2013; Zandalinas et al. 2020). The H_2_O_2_ sensor HYDROGEN-PEROXIDE-INDUCED Ca^2+^ INCREASES1, which is located on the guard cell PM, is responsible for the H_2_O_2_-responsive Ca^2+^ influx that initiates stomatal closure (Wu et al. 2020). The Ca^2+^ wave is a systemic signaling mechanism for defense (Lecourieux et al. 2006; Toyota et al. 2018). The Ca^2+^ channel REDUCED HYPEROSMOLALITY-INDUCED Ca^2+^ INCREASE1 functions in guard cells and roots, mediating osmotic stress–inducible Ca^2+^ influx in guard cells (Yuan et al. 2014). Liquid–liquid phase separation is another plausible mechanism for monitoring environmental changes (Eljebbawi et al. 2023). In the context of water availability, the prion-like protein FLOE1 stimulates seed germination in Arabidopsis through a reversible hydration-dependent phase separation process (Dorone et al. 2021).

In addition to directionless diffusion of signals, plants also use short peptides for directional signaling. The CLAVATA3/EMBRYO-SURROUNDING REGION-RELATED (CLE) family consists of mobile peptides that play active roles in development (Fletcher 2020). Thus, the discovery of a role for Arabidopsis CLE25 in drought responses was unexpected. Soil dehydration leads to CLE25 accumulation in roots, which then travels through the leaf vasculature (Fig. 3). At the leaf PM, the receptor-like protein kinases BARELY ANY MERISTEM1 (BAM1) and BAM3 perceive CLE25, inducing *NCED3* expression and ultimately resulting in stomatal closure (Takahashi et al. 2018). Notably, the Arabidopsis TF NGATHA1 mediates the drought-responsive induction of *NCED3* predominantly in the leaf vasculature (Sato et al. 2018). This coincidence suggests that CLE25–BAM ligand formation leads to ABA biosynthesis in leaves. Systematic studies have shown that most *CLE* family members are expressed in roots (Jun et al. 2010), encouraging further exploration of CLE-mediated root–shoot stress communication.

## Plant responses to cold stress

Cold stress has severe effects on plant growth and survival. Plant growth and development are negatively affected by chilling temperatures (0–12 °C) due to the inhibition of various vital biochemical reactions, such as decreases in water and nutrient uptake (Lyons 1973). Chilling stress also causes stomatal closure and the subsequent inhibition of photosynthesis and transpiration activity, with negative effects on plant growth similar to those of drought stress (Wilkinson et al. 2001). Furthermore, under freezing temperatures (below 0 °C), ice crystals start to form and damage cell membranes and cell walls. Ice nucleation occurs in intercellular spaces, leading to decreased water potential outside the cells. Consequently, water moves out of the cells to the intercellular space, resulting in severe cellular dehydration.

Plants acquire tolerance to subsequent freezing conditions through temporary exposure to chilling but nonfreezing temperatures. Cold acclimation involves dramatic transcriptional and metabolic changes that protect plant cells from cold-induced damage. These changes during cold acclimation include the accumulation of osmoprotectants and ROS scavenging, which also occur during drought stress responses (Cook et al. 2004; Maruyama et al. 2009; Mittler et al. 2022). Thus, some cold-responsive genes overlap with drought-responsive genes (Maruyama et al. 2012). In addition, cold stress alters the PM composition to mitigate decreased fluidity and freezing injury (Miki et al. 2019). Moreover, nonlethal freezing temperatures (approximately −3 °C) induce cell wall modifications and alter extracellular components, thereby inducing an additional response referred to as subzero acclimation (Takahashi et al. 2019, 2021).

### Transcriptional networks involved in cold-responsive gene expression

As described in the previous section, DREB1/CBF proteins, which are found only in angiosperms, function as major transcriptional regulators of cold stress responses by binding to the DRE/CRT elements in the promoters of their target genes (Stockinger et al. 1997; Liu et al. 1998; Shinwari et al. 1998). The Arabidopsis genome contains 3 *DREB1/CBF* genes, which are tandemly arrayed on chromosome 4: *DREB1B*/*CBF1*, *DREB1A/CBF3*, and *DREB1C/CBF2*. The constitutive expression of *DREB1/CBF* genes in transgenic plants upregulated hundreds of cold- and drought-responsive genes, even under normal growth conditions (Seki et al. 2001; Fowler and Thomashow 2002; Maruyama et al. 2004, 2009), improving tolerance to freezing, drought, and high-salinity stress (Jaglo-Ottosen et al. 1998; Liu et al. 1998; Kasuga et al. 1999). Many of the upregulated genes encode proteins necessary for cold stress tolerance and acclimation, such as LEA proteins, and osmolyte biosynthesis enzymes. Disruption of all 3 *DREB1/CBF* genes by genome editing resulted in dramatically reduced cold-inducible gene expression and tolerance of freezing stress (Jia et al. 2016; Zhao et al. 2016). By contrast, overexpressing *DREB1/CBF* led to the downregulation of growth-related genes and adversely affected plant growth (Kasuga et al. 1999; Sakamoto et al. 2004; Achard et al. 2008; Kudo et al. 2019).

### Upstream regulators of cold-responsive *DREB1/CBF* expression

Three *DREB1/CBF* genes in Arabidopsis are rapidly induced by cold stress (Liu et al. 1998; Shinwari et al. 1998). Several TFs have been reported to regulate *DREB1/CBF* expression (Thomashow 2010; Kidokoro et al. 2022). The MYC-like basic helix-loop-helix (bHLH) TF INDUCER OF CBF EXPRESSION1 (ICE1) was identified following the isolation of the Arabidopsis *ice1-1* mutant in a screen for mutations that impair the cold-induced transcription of the firefly luciferase (*LUC*) reporter gene driven by the Arabidopsis *DREB1A/CBF3* promoter (Chinnusamy et al. 2003). In the *ice1-1* mutant, cold-induced expression of endogenous *DREB1A/CBF3* was reduced, whereas *DREB1B/CBF1* and *DREB1C/CBF2* expression was unaffected. It was long believed that ICE1 promotes cold stress tolerance by directly activating *DREB1A/CBF3*. However, *DREB1A/CBF3* repression in *ice1-1* plants is not caused by the mutation originally identified in ICE1 (R236H); rather, it is due to DNA methylation–mediated gene silencing triggered by the inserted T-DNA harboring the *DREB1A/CBF3pro:LUC* reporter construct. Therefore, ICE1 does not regulate *DREB1A/CBF3* expression in response to cold stress (Kidokoro et al. 2020). Nevertheless, ICE1 is identical to SCREAM (SCRM), which regulates stomatal development along with its homolog SCRM2; the *ice1-2 scrm2-1* double mutant exhibits an epidermis devoid of stomata (Kanaoka et al. 2008). The dominant *SCRM* mutant allele *scrm-D* carries the same missense mutation (R236H) in SCRM/ICE1 as *ice1-1*, and both mutants present clear defects in stomatal development (Kanaoka et al. 2008). However, cold stress–induced *DREB1A/CBF3* expression did not decrease in *scrm-D* mutants compared with wild-type plants (Kidokoro et al. 2020). This finding validates the idea that *DREB1A/CBF3* repression in *ice1-1* is not caused by the ICE1^R236H^ mutation and that ICE1 is not involved in cold stress–responsive *DREB1A/CBF3* expression. There have been many reports on the effect of *ICE1* overexpression on cold stress tolerance in crops, but they need to be carefully reassessed (Kidokoro et al. 2020; Thomashow and Torii 2020).

Other TFs are also crucial regulators of *DREB1/CBF* expression during early cold stress responses, including CALMODULIN-BINDING TRANSCRIPTION ACTIVATOR (CAMTA) TFs and circadian clock–related MYB-like TFs such as REVEILLEs (RVEs), CIRCADIAN CLOCK ASSOCIATED1 (CCA1), and LATE ELONGATED HYPOCOTYL (LHY) (Doherty et al. 2009; Kidokoro et al. 2017, 2021). CAMTAs are CG-1-type TFs that are conserved in plants and other multicellular eukaryotes (Bouché et al. 2002). These proteins contain calmodulin-interacting domains: IQ motifs and a calmodulin-binding domain. The CAMTA TFs recognize the CGCG-box [(A/C/G)CGCG(G/T/C)] and strongly upregulate *DREB1B/CBF1* and *DREB1C/CBF2* (Kidokoro et al. 2017). Unlike CAMTA TFs, circadian clock–related MYB-like TFs bind to a *cis*-acting motif known as the evening element (EE) [AAAATATCT] in the *DREB1A/CBF3* and *DREB1C/CBF2* promoters (Kidokoro et al. 2021). Detailed analysis of these CAMTA and MYB-like TFs revealed that Arabidopsis recognizes cold stress as 2 different signals (rapid or gradual decreases in temperature) and that the expression of *DREB1A/CBF3* genes is differentially induced by these 2 stress signals through 2 different signal transduction pathways (Kidokoro et al. 2017). The CAMTA TFs only respond to a rapid drop in temperature, but the MYB-like TFs recognize both rapid and gradual decreases in temperature. The MYB-like TFs strongly induce *DREB1/CBF* expression in response to cold only in the daytime; by contrast, CAMTA TFs activate *DREB1/CBF* expression regardless of the time of day (Kidokoro et al. 2017).

### CAMTA-mediated regulatory pathways

**Figure 4.**
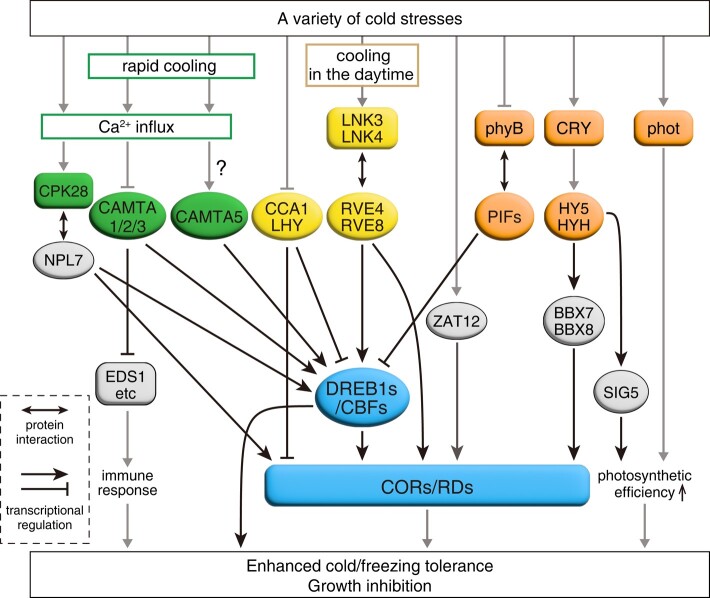
The regulatory network activated under cold stress conditions. In angiosperms, DREB1/CBF transcription factors regulate cold acclimation and stress tolerance acquisition by inducing numerous *COR* and *RD* genes. CAMTA and RVE transcription factors rapidly induce *DREB1/CBF* expression. By contrast, CCA1, LHY, and PIFs repress *DREB1/CBF* expression under normal growth conditions. CAMTA3 is also involved in plant immune responses and inhibits growth under cold stress conditions together with CAMTA1 and CAMTA2. Other transcription factors, such as NPL7, HY5, HYH, and ZAT12, also promote cold stress tolerance. Moreover, several photoreceptors, such as phyB and phot, influence cold stress responses. Notably, phototropins in liverwort perceive cold stress and regulate chloroplast relocation. Circles indicate transcription factors. Yellow, orange, and green represent proteins involved in the circadian clock, light signaling, and calcium (Ca^2+^) signaling, respectively.

Among Arabidopsis CAMTA family members (CAMTA1–6), CAMTA3 (also named *Arabidopsis thaliana* SIGNAL-RESPONSIVE GENE1), along with its homologs CAMTA1 and CAMTA2, activate *DREB1B/CBF1* and *DREB1C/CBF2* expression (Doherty et al. 2009; Kim et al. 2013) (Fig. 4). These CAMTA proteins also act as transcriptional repressors of plant immune signaling (Du et al. 2009). This signaling is induced not only by pathogen infection but also by long-term low-temperature conditions and by mechanical stimuli such as raindrops (Kim et al. 2013, 2020; Matsumura et al. 2022). The CAMTA-mediated pathway is likely involved in Ca^2+^ signaling, as these TFs contain calmodulin-binding sites (Bouché et al. 2002). However, the N-terminal 334–amino acid region of CAMTA3, which contains the CG-1 DNA-binding domain and lacks calmodulin-binding sites, is sufficient to induce *DREB1/CBF* gene expression in response to cold stress and to suppress immune-related genes under normal temperatures (Kim et al. 2017; Chao et al. 2022). Under cold stress conditions, calmodulin-binding sites in CAMTA3 release their negative regulation of the N-terminal region of this protein, leading to immune-responsive gene expression (Kim et al. 2017). The CG-1 DNA-binding domain of CAMTA3 modulates its transcriptional activity in response to cold, whereas CAMTA3 stably binds to target promoters under both unstressed and cold stress conditions (Chao et al. 2022). Therefore, the role of the calmodulin-binding sites of CAMTA3 in cold stress–induced transcriptional regulation remains unclear.

In addition to CAMTA3, an analysis of quintuple and sextuple *camta* mutants revealed that CAMTA5 also induces *DREB1/CBF* expression (Kidokoro et al. 2017). CAMTA5 functions in the cold stress response but not in the immune response, whereas CAMTA3 regulates both cold stress and immune responses. Moreover, these CAMTA proteins regulate different target genes during cold stress (Chao et al. 2022), indicating that although both CAMTA3 and CAMTA5 activate *DREB1/CBF* expression, their activation mechanisms may differ. Additional analyses are required to elucidate the Ca^2+^ signaling–regulated functions of CAMTA proteins in cold-responsive gene expression.

### Clock-related TF-mediated regulatory pathways

In the Arabidopsis circadian clock, several transcriptional repressors form negative feedback loops and are key oscillators under normal growth conditions. These repressors are CCA1 and LHY, PSEUDO-RESPONSE REGULATORs (PRR9, PRR7, PRR5, and PRR1/TIMING OF CAB EXPRESSION1 [TOC1]), and an evening complex (EC) that consists of LUX ARRHYTHMO (LUX), EARLY FLOWERING3 (ELF3), and ELF4 (Nohales and Kay 2016). CCA1 and LHY levels rise shortly before dawn; these TFs repress evening-phased genes via the evening element (EE) mentioned above and the CCA1-binding site (Nagel et al. 2015; Kamioka et al. 2016). Three RVE proteins, RVE4, RVE6, and RVE8, then activate these genes in the evening (Hsu et al. 2013).

The regulation of cold-inducible gene expression by circadian clock–related MYB-like TFs resembles circadian-controlled gene expression. CCA1 and LHY repress cold-inducible gene expression under normal temperatures, whereas RVEs activate cold-responsive genes under cold stress. RVE4 and RVE8 play dominant roles in cold-inducible gene expression (Kidokoro et al. 2021). RVE6 redundantly and conditionally activates its target cold-inducible genes together with RVE3 and RVE5, when *RVE4* and *RVE8* are not expressed. CCA1, LHY, RVE4, and RVE8 activities are controlled by post-translational modifications. Under normal temperature conditions, CCA1 and LHY localize to the nucleus, whereas RVE4 and RVE8 are located mainly in the cytosol and partly in the nucleus (Kidokoro et al. 2021). During the early cold stress response, CCA1 and LHY are degraded, but RVE4 and RVE8 quickly accumulate in the nucleus to activate cold-inducible transcription.

Truncation of potential PEST sequences in CCA1 prevented its degradation and led to decreased *DREB1A/CBF3* induction under cold stress. This finding suggests that CCA1 and LHY degradation is important for cold-inducible gene expression, in addition to the nuclear accumulation of RVE4 and RVE8. Additionally, RVE4 and RVE8 activate heat-inducible genes such as *ETHYLENE RESPONSE FACTOR53* (*ERF53*) and *ERF54* and regulate heat stress tolerance during the day (Li et al. 2019). Taken together, these findings indicate that RVE4 and RVE8 likely activate distinct sets of target genes through different signaling pathways under normal temperatures or cold or heat stress conditions. In response to heat, RVE4 and RVE8 accumulate in the nucleus (Kidokoro et al. 2021). However, *DREB1/CBF* expression is not induced by heat stress, indicating that factors other than the nuclear accumulation of RVE4 and RVE8 are also required to regulate target genes under each temperature condition.

Under normal growth conditions, NIGHT LIGHT-INDUCIBLE AND CLOCK-REGULATED1 (LNK1) and LNK2 interact with RVEs. LNK1 and LNK2 act as RVE coactivators by recruiting the transcriptional machinery to target RVEs to the promoters of circadian-controlled genes (Rugnone et al. 2013; Xie et al. 2014; Ma et al. 2018). Similarly, LNK proteins also function as coactivators in stress-inducible gene expression (Kidokoro et al. 2023). Among the 4 Arabidopsis LNK proteins, LNK3 and LNK4 regulate cold-inducible gene expression. Because RVE4, RVE8, and LNK proteins accumulate in the nucleus during the day, many cold-inducible target genes, including *DREB1/CBF* genes, are strongly induced by cold stress in the daytime (Kidokoro et al. 2021, 2023). Furthermore, LNK3 and LNK4 are specifically phosphorylated under cold conditions, whereas RVEs and LNKs stably interact under all temperature conditions. Therefore, the specific phosphorylation of LNKs may control cold-specific target gene expression. However, LNK1 and LNK2 function under heat stress in addition to normal temperature conditions (Kidokoro et al. 2023). The heat stress response pathway mediated by RVEs–LNK1/LNK2 is independent from another major pathway regulated by the HEAT SHOCK FACTOR1A family (Ohama et al. 2016).

Moreover, PRR proteins recognize the *DREB1/CBF* gene promoters and regulate their expression. The triple loss of function of *PRR9*, *PRR7*, and *PRR5* resulted in the strong upregulation of *DREB1/CBF* genes and the downstream genes of their encoded TFs under normal growth conditions (Nakamichi et al. 2009, 2012). These findings indicate that multiple circadian clock–related TFs regulate *DREB1/CBF* expression.

### Other transcription factors regulating the cold stress response

Several TFs that are key regulators of other signaling pathways also control *DREB1/CBF* expression, indicating that the regulation of *DREB1/CBF* genes is complex (Fig. 4) (Kidokoro et al. 2022). PHYTOCHROME-INTERACTING FACTORs (PIFs) contain a putative phytochrome-interacting domain and are involved in photomorphogenesis (Leivar and Quail 2011). Among these bHLH TFs, Arabidopsis PIF3, PIF4, and PIF7 and rice OsPIL16 negatively regulate *DREB1* expression, whereas tomato (*Solanum lycopersicum*) SlPIF4 enhances *DREB1/CBF* expression and cold acclimation (Kidokoro et al. 2009; Lee and Thomashow 2012; Jiang et al. 2017; Wang et al. 2020a). bZIP-type TFs, including ELONGATED HYPOCOTYL5 (HY5), are key components of light signaling. HY5 stability is regulated by E3 ubiquitin ligase complexes, including CONSTITUTIVELY PHOTOMORPHOGENIC1, in a blue light–dependent manner. Under cold stress conditions, HY5 is stabilized and upregulates target genes encoding B-BOX DOMAIN PROTEIN TFs and anthocyanin biosynthesis enzymes (Catalá et al. 2011; Li et al. 2021). HY5 and HY5 HOMOLOG (HYH) also induce *SIGMA FACTOR5* (*SIG5*) expression in a circadian manner (Cano-Ramirez et al. 2023). SIG5 regulates the expression of chloroplast genes such as *psbD* to maintain photosynthetic efficiency under long-term cold stress. NIN-LIKE PROTEIN7, a key TF involved in nitrate responses, activates the cold-inducible expression of *DREB1/CBF* and their downstream genes (Ding et al. 2022). Moreover, cold stress activates the PM-localized CALCIUM-DEPENDENT PROTEIN KINASE28 (CPK28) in a Ca^2+^-dependent manner. CPK28 induces a phosphorylation cascade that triggers the translocation of NPL7 into the nucleus (Ding et al. 2022).

In addition to the DREB1/CBF-mediated pathway, other pathways are also involved in the acquisition of cold tolerance through transcriptional regulation. In particular, the clock-related factors RVE4, RVE8, CCA1, and LHY directly bind to the EE and regulate many *RD* (*RESPONSIVE TO DEHYDRATION*) and *COR* genes (Kidokoro et al. 2021). The EE is enriched in the promoters of cold-inducible genes in several plant species, such as Arabidopsis, soybean, and rice (Mikkelsen and Thomashow 2009; Maruyama et al. 2012). ZINC FINGER OF ARABIDOPSIS THALIANA12 is another TF that upregulates *RD* and *COR* gene expression, whereas it downregulates *DREB1/CBF* expression (Vogel et al. 2005). COR27 and COR28 are negative regulators of freezing tolerance, but their encoding genes are transcriptionally induced by cold (Li et al. 2016). They repress the transcription of evening phase genes such as *TOC1* by interacting with and destabilizing the RVE8–LNK1/LNK2 complex (Sorkin et al. 2023). The existence of various transcriptional regulatory pathways for cold-inducible gene expression reflects the ability to acclimate to complex cold stress conditions in nature.

### Perception of cold stress

Although many TFs involved in cold stress signaling have been reported, how plants perceive temperature changes and adapt to different climates remain poorly understood (Verslues et al. 2023). Ca^2+^ is a general second messenger involved in various signaling pathways. While plants lack orthologs of transient receptor potential cation channels, which are well-known thermosensors in many organisms, including mammals (Kashio and Tominaga 2022), Ca^2+^ signaling plays a crucial role in cold stress responses, including in the DREB1/CBF-dependent and -independent pathways (Plieth et al. 1999; Yuan et al. 2018).

Cold stress triggers rapid and transient increases in cytosolic Ca^2+^ levels in a cooling rate–dependent and/or tissue-specific manner in plants (Knight and Knight 2000; Hiraki et al. 2019; Lamers et al. 2020). Several PM-localized proteins, such as calcium-permeable mechanosensitive channels (MCA1 and MCA2), cyclic nucleotide-gated ion channels, and Ca^2+^-permeable transporters (ANNEXINs), mediate increases in cytosolic Ca^2+^ levels under cold stress (Mori et al. 2018; Cui et al. 2020; Liu et al. 2021). In rice, the PM and endoplasmic reticulum–localized protein CHILLING-TOLERANCE DIVERGENCE1 activates Ca^2+^ influx in response to cold stress and induces cold stress tolerance by regulating G-protein signaling (Ma et al. 2015). In cold stress–activated Ca^2+^ signaling pathways, Ca^2+^/calmodulin-regulated receptor-like kinases and Ca^2+^-dependent protein kinases (CPKs/CDPKs) positively regulate cold tolerance by activating MAP kinase cascades (Yang et al. 2010a, 2010b; Yuan et al. 2018). In maize, ZmMPK8 phosphorylates type-A Response Regulator1, leading to its degradation and the induction of cold-responsive gene expression to promote chilling tolerance (Zeng et al. 2021).

Several photoreceptors function as thermosensors in plants (Noguchi and Kodama 2022; Jung et al. 2023; Kerbler and Wigge 2023). The red-light photoreceptor PHYTOCHROME B (phyB) regulates photomorphogenesis, light-mediated development, and thermomorphogenesis under normal temperatures in Arabidopsis (Legris et al. 2016; Jung et al. 2023). While red light stimulates conversion between 2 photo-interconvertible forms of phyB, from the inactive Pr state to the active Pfr state, cool temperatures prevent the reversion of phyB from the Pfr state to the Pr state. In Arabidopsis and several other plants, light quality and photoreceptors such as phyB affect cold acclimation and freezing tolerance (Kim et al. 2002; Franklin and Whitelam 2007; Wang et al. 2016; Prerostova et al. 2021). In the liverwort *Marchantia polymorpha*, the blue light receptor phototropin (phot) regulates chloroplast positioning under low-temperature conditions via the temperature-dependent lifetime of its photoactivated chromophore (Fujii et al. 2017). Among the central oscillators of the Arabidopsis circadian clock, the EC functions as a thermosensor. ELF3, an EC component that contains a polyglutamine-rich repeat embedded within a prion-like domain, is a large scaffold protein and a key temperature-sensing component (Jung et al. 2020). ELF4, another EC component, strengthens the DNA-binding activity of the EC at low temperatures (Ezer et al. 2017; Silva et al. 2020). In addition, ELF4 can be transported from shoots to roots to deliver circadian information; low temperatures promote its mobility (Chen et al. 2020). However, how the EC perceives the cold signal and functions at temperatures below 12 °C remain unknown.

## Interconnection between drought and cold responses

Exogenous ABA application improves the plant cold tolerance, while cold stress alone only causes a modest increase in ABA levels compared with drought stress (Maruyama et al. 2012). Both stresses lead to stomatal closure, but during cold stress, this mechanism appears to be independent of ABA (Wilkinson et al. 2001). ABA-deficient or -insensitive mutants exhibit reduced freezing stress tolerance, although the cold-inducible expression of *COR* and *RD* genes may not always be affected in these mutants, indicating that a substantial part of the cold response operates independently of ABA (Gilmour and Thomashow 1991). Therefore, ABA contributes to plant cold resilience through its cumulative effects on growth and development, alongside its role in signaling during the drought response (Yoshida and Fernie 2023).

DREB1/CBF and DREB2 TFs are highly homologous isoforms that share the DRE/CRT *cis*-acting element for identifying their target genes. This trait positions them as a pivotal interconnection point in gene regulation of drought and cold stress responses. DREB2 homologs have been widely identified in both mosses and lycophytes as essential components of drought stress signaling. By contrast, DREB1/CBF homologs have been identified exclusively in angiosperms, highlighting a significant evolutionary adaptation (Zhang et al. 2004; Ito et al. 2006; Mizoi et al. 2012; Kidokoro et al. 2015; Li et al. 2020). This specialization suggests that the regulation of cold-responsive genes in plants has evolved to cope with the challenges posed by habitat expansion onto dry land, where plants face reduced water availability and do not receive the temperature buffering provided by aquatic environments. As a result, plants have developed diverse sensing mechanisms that converge on a shared set of protective genes, enabling them to effectively respond to both the combined and specific environmental challenges of cold and drought.

## Breeding stress-resilient crops

Improving stress tolerance is a major focus of plant stress biology and crop breeding programs, with many significant advancements already documented (Bailey-Serres et al. 2019; Cooper and Messina 2023). Specifically, in molecular breeding, a range of stress-related genes have been incorporated into transgenic crops to determine their effects on stress resilience during field trials. Targeting the ABA receptor to adjust stress sensitivity via the ectopic expression of PYR1/PYL/RCAR homologs in Arabidopsis and wheat (*Triticum aestivum*) enhanced drought tolerance and water use efficiency with minimal yield losses (Yang et al. 2016; Mega et al. 2019). However, transgenic rice expressing these homologs showed reduced growth under field conditions (Kim et al. 2014). Another strategy for improving tolerance is metabolic engineering, such as the introduction of Arabidopsis GolS2 into rice, which increased osmoprotectant levels and maintained yields under drought (Selvaraj et al. 2017). Stress-signaling TFs, such as DREB1/CBF and its homologs, have been successfully used to increase drought tolerance in crops including wheat, soybean, and rice (Xiao et al. 2009; Saint Pierre et al. 2012; Fuganti-Pagliarini et al. 2017). Although evaluating cold tolerance in field trials is challenging, overexpressing rice *bZIP73* notably improved cold tolerance in rice at the reproductive stage (Liu et al. 2019). Additionally, the overexpression of various fatty acid desaturase genes enhanced cold stress tolerance in crops, with some demonstrating field-level cold resilience (Dar et al. 2017). Commercially available transgenic maize expressing bacterial cold shock protein, which acts as an RNA chaperone, showed improved environmental resilience and yield retention (Nemali et al. 2015).

Recent biotechnological advancements allow for precise and flexible gene modulation and the ability to edit multiple genes simultaneously (Chen et al. 2019; Shi et al. 2023). Therefore, future molecular breeding efforts are expected to involve physiological context-based strategies, moving from the current single-gene interventions (Husaini 2022). For instance, enhancing drought tolerance may involve a variety of changes, such as increasing ABA sensitivity, augmenting leaf wax layers, and reducing stomatal density to conserve water. Similarly, for cold tolerance, adjusting the switch between growth and stress responses through CAMTA- and circadian clock–regulated pathways may offer improvements.

## New challenges and perspectives

New technologies have the potential to expand the field of plant stress biology. Recent advancements have ushered in the capability for genome-wide analyses of the transcriptome and epigenome at the single-cell level, granting researchers the ability to dissect the molecular profiles of individual cells implicated in abiotic stress responses, such as guard cells and phloem companion cells (Shaw et al. 2020; Seyfferth et al. 2021). Spatial sequencing is poised to enhance our understanding of single-cell data within the framework of intercellular positioning (Giacomello 2021). Additionally, computational approaches empowered by machine learning are refining the analysis of nuanced developmental changes under complex environmental conditions, complemented by automated phenotyping (Fujita et al. 2018; Lew et al. 2020; Singh et al. 2021). Advanced imaging methods such as hyperspectral imaging and super-resolution microscopy are providing unprecedented spatial and temporal insights into plant stress reactions, enabling a detailed exploration of variability in stress responses and the signaling pathways that mediate stress adaptation across different genotypes and environmental conditions (Galieni et al. 2021; Ovečka et al. 2022). These technological developments are instrumental for researchers seeking to decode the complex networks of plant stress responses and to engineer crops with optimized traits for stress resilience.

## Dive Curated Terms

The following phenotypic, genotypic, and functional terms are of significance to the work described in this paper:

ABI5 Gramene: At2g36270
ABI5 Araport: At2g36270

ABI1 Gramene: AT4G26080
ABI1 Araport: AT4G26080

ELF4 Gramene: AT2G40080
ELF4 Araport: AT2G40080

MCA2 Gramene: AT2G17780
MCA2 Araport: AT2G17780

MYB2 Gramene: AT2G47190
MYB2 Araport: AT2G47190

AHG1 Gramene: AT5G51760
AHG1 Araport: AT5G51760

HAB2 Gramene: AT1G17550
HAB2 Araport: AT1G17550

PIF3 Gramene: AT1G09530
PIF3 Araport: AT1G09530

PIF4 Gramene: AT2G43010
PIF4 Araport: AT2G43010

PIF7 Gramene: AT5G61270
PIF7 Araport: AT5G61270

MYC2 Gramene: AT1G32640
MYC2 Araport: AT1G32640

PRR7 Gramene: AT5G02810
PRR7 Araport: AT5G02810

RBOHD Gramene: At5g47910
RBOHD Araport: At5g47910

ABI2 Gramene: AT5G57050
ABI2 Araport: AT5G57050

WRKY18 Gramene: AT4G31800
WRKY18 Araport: AT4G31800

DREB2A Gramene: AT5G05410
DREB2A Araport: AT5G05410

RD22 Gramene: AT5G25610
RD22 Araport: AT5G25610

CBF1 Gramene: AT4G25490
CBF1 Araport: AT4G25490

CAMTA3 Gramene: AT2G22300
CAMTA3 Araport: AT2G22300

NAC CHEBI: CHEBI:7421
